# The effects of personality traits and attitudes towards the rule on academic dishonesty among university students

**DOI:** 10.1038/s41598-022-18394-3

**Published:** 2022-08-19

**Authors:** Hongyu Wang, Yanyan Zhang

**Affiliations:** Department of Psychology, School of Philosophy and Sociology, Jilin University, Changchun, China

**Keywords:** Psychology, Human behaviour

## Abstract

Academic dishonesty is becoming a big concern for the education systems worldwide. Despite much research on the factors associated with academic dishonesty and the methods to alleviate it, it remains a common problem at the university level. In the current study, we conducted a survey to link personality traits (using the HEXACO model) and people’s general attitudes towards the rule (i.e., “rule conditionality” and “perceived obligation to obey the law/rule”) to academic dishonesty among 370 university students. Using correlational analysis and structural equation modeling, the results indicated that both personality traits and attitudes towards the rule significantly predicted academic misconduct. The findings have important implications for researchers and university educators in dealing with academic misconduct.

## Introduction

Academic dishonesty is a global issue that attracts much attention from educators worldwide, and relevant research could date back to the last century^1^. It is considered immoral and inappropriate because the behavior has an unfair advantage over other students and impedes individuals’ capacity to study^2,3^. Dishonesty behavior often starts early in school, such as copying others’ work, and has been a consistent and paramount problem throughout all education levels^4^. It is an educational and academic issue with severe consequences^5^. Engagement in academic dishonesty predicts increased acceptance of immoral workplace behavior, indicating its continuous influence post-graduation^6,7^. At the university level, such misconduct behavior has clear potential to diminish the reputation and integrity of universities. It hinders universities’ ability to ensure that students who achieve degrees have the knowledge and skills they require for employment or further study^8^.

Much literature on the individual predictors of academic cheating has mostly focused on the influences of personality traits, academic attitudes & values, and some demographic variables^9^. Several personality traits were found to be significantly predictive, such as impulsivity^10^, psychopathy^11^, Machiavellianism and narcissism^12^. Self-control may explain why people do, or do not, engage in plagiarism when the opportunity is available^13^. Curtis et al.^14^ found that self-control and academic misconduct were negatively correlated. Although individuals’ self-control can vary depending on situational factors such as mood, fatigue, and hunger, it is more like stable personality-like differences^15^.

Research on the demographic variables found that age and gender are critical factors predicting academic misconduct. For instance, as they age, female college students are less likely to engage in academic dishonesty due to fewer comparisons between one’s own behaviors and peer behaviors^16^. However, age and gender are not consistently found to be significant factors in most research on academic misconduct^9,17^.

In the current study, we still focused on these individual factors with attempts to 1) assess a recent model of personality (i.e., HEXACO) and its predictive power of academic dishonesty; and 2) to link people’s general attitude towards the rule/law to academic dishonesty, considering that it is essentially a violation of the rules in academia. Age and gender effects are examined along with the above goals.

### Academic dishonesty

Academic dishonesty, academic misconduct, academic cheating, and academic integrity are concepts often used interchangeably in previous literature. The concept is usually defined through behavioral classifications. Pavela^18^ considered academic dishonesty to contain four main types of misconduct that deliberately violate school regulations: cheating, fabrication, facilitation, and plagiarism. McCabe and Trevino^19^ further expanded the scope of this concept into 12 types of violating behaviors in school, including sneaking at notes in the exam, copying others’ answers in the exam, copying others’ answers without their permission in the exam, etc. These researchers developed a 12-item scale to measure academic dishonesty, which is widely used^19,20^. However, Adesile and Nordin^21^ criticized that the psychometric properties have not been critically investigated despite their broader literature application. To validate the psychometric properties of the instrument and determine the dimensionality of academic dishonesty, Adesile^21^ adapted the original scale, divided academic dishonesty into three dimensions (“cheating,” “plagiarism,” and “research misconduct”), and named it “the academic integrity survey.”

Academic dishonesty is found to be largely explained by individual factors^22^. Motivation is one of the major individual factors relating to academic dishonesty. Through a meta-analytic investigation, Krou and colleagues^23^ reviewed 79 studies and reported that academic dishonesty was negatively associated with intrinsic motivation, self-efficacy, utility value, and internal locus of control, and was positively associated with amotivation and extrinsic goal orientation. Motivation, however, may vary across different cultural backgrounds. A recent study of Chinese university students found that their unethical academic behaviors are associated with their unique motivation to meet parents’ expectations^24^.

Morality is another crucial predictor of academic cheating and plagiarism. Individuals with a high level of morality, emphasis on fairness, and value of social rules have stringent attitudes towards plagiarism^25–27^. Meanwhile, moral disengagement is positively associated with cheating^28^. The predicting effect of morality may also be inconsistent across cultures. Ampuni et al.^29^ studied the relationship between academic dishonesty and the five moral foundations in Indonesia, and found only a weak predictive power of the “authority” foundation on academic dishonesty.

Since the COVID-19 epidemic outbreak, courses and examinations have been conducted online, and researchers are concerned that academic misconduct in online learning environments has become more serious^30^. Studies, however, provided little and even opposite evidence regarding the actual behavioral differences in academic misconduct between traditional and online settings. Peled et al.^31^ found that students tend to engage less in academic dishonesty behaviors online than in face-to-face courses. In addition, cheating intentions among students in traditional and online education settings are very little^32^. To reconcile the inconsistent findings, researchers started considering potential moderating and mediating factors such as the types of academic dishonesty, the level of technology complexity, and statistics anxiety^33–35^.

### Personality traits and academic dishonesty

Personality reflects a person’s consistent patterns of thoughts, feelings, and behaviors. It has a large effect on individuals’ academic behaviors. The Big Five personality model is the most widely used predictor of academic dishonesty. For instance, Giluk and Postlethwaite^36^ reviewed studies of both high school and university students, and concluded that conscientiousness and agreeableness (of the Big Five) are the strongest predictors of academic dishonesty. Graziano and Eisenberg^37^ found agreeable people more trusting and less cynical. As a result, they were less likely to justify cheating and see it as a necessity to compete with others. Lee et al.^9^ conducted a meta-analysis on predictors of academic dishonesty and confirmed the strong relationship between agreeableness and academic dishonesty. They also found openness to be associated with self-efficacy/personal ability, and in turn, was negatively related to academic dishonesty. Finally, a positive association between neuroticism and academic procrastination increased cheating behaviors at school^38^.

Empirical evidence on the relationship between extraversion and academic dishonesty, however, is not consistent. For example, some research found a small positive association between extraversion and scholastic dishonesty^11^, while others indicated a moderate negative association^39^, and nonsignificant findings^36^.

Ashton and Lee^40^ extended the Big Five personality model with a set of lexical studies, and developed a six-dimension model, referred to as the HEXACO model of personality structure. The name of this model reflects both the number of factors (i.e., the Greek *hexa*, six) and their names: Honesty-Humility (H), Emotionality (E), extraversion (X), Agreeableness (A), Conscientiousness (C), and Openness to Experience (O). It is essential the Big Five personality plus an additional Honesty-Humility dimension. The six-dimensional structure was more replicable across cultures than the Big Five model, as the Big Five structure has failed to present in four languages that recovered the HEXACO dimensions^41^. It has become a major tool in measuring personality traits in the early 21st century.

The HEXACO model, particularly the Honesty-Humility (H) dimension, is proven to be very useful in predicting many unethical behaviors. Kleinlogel et al.^42^ investigated the relationship between Honesty-Humility and cheating behavior. Results showed that individuals high in Honesty-Humility were less likely to cheat than those low on this trait. Honesty-Humility was negatively associated with adolescents’ unethical behavior, and moral disengagement partially mediated this negative association^43^. Hilbig and Zettler^44^ found that German adults who were low in Honesty-Humility were more likely to behave dishonestly across various experimental situations (e.g., coin-toss task and dice-task). Honesty-Humility was negatively associated with unethical business decisions among people from Fiji and the Marshall Islands^45^. Honesty-Humility was proved to be the strongest predictor of cheating, dishonesty, counterproductive behavior, and antisocial behavior, according to a meta-analysis^41^. Accordingly, the current study posits that,

#### Hypothesis 1

All six dimensions of the HEXACO model will predict academic dishonesty. Specifically, Honesty-Humility (H), Agreeableness (A), Conscientiousness (C), Openness to Experience (O), and Emotion Stability (E) are all expected to predict academic dishonesty negatively.

### Attitudes towards the rule: perceived obligation of rule/law and rule conditionality

The concept of the perceived obligation of law/rule proposed (short for POOL) by Tyler^46^ refers to individuals’ variability in perceptions of obeying general laws. The higher the endorsement, the more likely they are to comply with laws and rules. If one’s POOL level is high, it has nothing to do with the fear of violating and thus being punished by the laws, and it is also not because someone sees other people’s compliance behaviors and tries to conform and comply. POOL exists at the personal level, which arises from people’s knowledge and conscience to stand up for the laws and rules.

Tyler’s research has shown a negative link between POOL and general criminal behavior. The more one perceives an obligation to obey the law, the less likely one will violate the law^47^. In other words, if one’s POOL is higher, one is not expected to perform academic misconduct, as the person would like to obey the rule or law, and volunteer to restrain one’s behavior. POOL is also found to be a key element in predicting compliance behavior to slow the spread of the virus during the COVID-19 pandemic^48^.

Rule conditionality (RC), also called rule orientation, assess the extent to which an individual perceives it is acceptable to violate the legal rules under certain conditions^49^. In other words, less rule-oriented people accept more reasonable circumstances to break the rules, and those who are more rule-oriented acknowledge fewer acceptable circumstances to violate the regulations.

RC derives from POOL and negatively relates to POOL, but they are very different. RC presents the level of flexibility when people evaluate different circumstances to break the law or rules. POOL is a sense of one’s obligation and duty to obey the laws and regulations. RC played a crucial role in predicting compliance behaviors and law violations. When laws go against personal morals, people will weigh the advantages, and disadvantages of immoral behavior, combined with moral belief, the lack of knowledge of the law, cost-benefit analysis, social norms, and lack of procedural justice are all critical roles in influencing the possibility of violating the law^49^.

In general, since both POOL and RC are stable personality-like variables that do not vary across mood and situations, and because misconducts in academia are rule violations by their nature (although the consequences are not similarly severe as law violations), we expect that students’ perception of the duty to obey the law/rule and their sense of rule conditionality would both be significant predictors of academic dishonesty. Accordingly, the current study expects that***,***

#### Hypothesis 2

RC positively predicted academic dishonesty, and POOL negatively predicted academic dishonesty. That is, participants who are more likely to consider rules as conditional, will report more cheating behaviors. In contrast, participants who perceive more obligation to obey the law, will report fewer cheating behaviors.

## Method

The current study has been approved by the IRB of School of Philosophy and Sociology of Jilin University. Informed consent has been obtained from all participants of the present study. All methods were performed in accordance with relevant guidelines and regulations.

### Sample

The sample consists of 370 students coming from a Northern Chinese University. The mean age of the participants was 19.77 years old (SD = 3.63). 224 (60.54%) participants majored in sciences and 146 (39.46%) participants majored in humanities and social sciences. 211 (57.03%) were male students, and 159 (42.97%) were female students.

### Instruments

All research instruments were originally in English, translated into Chinese by a psychology graduate student, and back-translated by a bilingual psychology researcher.

#### Academic dishonesty (AD)

The Academic Integrity Survey (AIS)^50^ was used to measure academic dishonesty (α = 0.916). It contains 15 items assessing the “Cheating,” “Research Misconduct,” and “Plagiarism” of academic misconduct. The survey comprised an 8-point Likert scale (1 = ‘very strongly disagree’; 8 = ‘very strongly agree’). A higher score on the scale indicated a higher level of academic dishonesty. The internal consistency for the whole scale in current study was 0.955, with Cronbach’s alpha being 0.919, 0.812, and 0.817 for “Cheating,” “Research Misconduct,” and “Plagiarism,” respectively.

#### Personality

The HEXACO model^40^, a six-dimension structure containing the factors Honesty-Humility (H), Emotionality (E), Extraversion (X), Agreeableness (A), Conscientiousness (C), and Openness to Experience (O), was used as a measure of personality, with 60 items in total. The internal consistency reliabilities ranged from 0.77 to 0.80 in the college sample and from 0.73 to 0.80 in the community sample^51^. The HEXACO-60 comprised a 5-point Likert scale (1 = ‘strongly disagree’; 5 = ‘strongly agree’). Cronbach’s alpha was 0.795(α_HH_ = 0.701, α_EX_ = 0.748, α_EM_ = 0.642, α_AG_ = 0.649, α_CO_ = 0.609, α_OP_ = 0.668) in the current study.

#### Rule conditionality (RC)

Rule Conditionality Scale^49^ was utilized to indicate the extent to which individuals perceive acceptable conditions for breaking the law in general (α = 0.928). The 7-point Likert scale (1 = ‘strongly disagree’; 7 = ‘strongly agree’) contains 12 items, which is calculated as a mean score (*M* = *3.49*, *SD* = 1.08), with higher scores indicating more rule conditionality (i.e., the individual accepts fewer justifications for violating laws). Cronbach’s alpha was 0.868 in the current study.

#### Perceived obligation to obey the law (POOL)

Perceived Obligation to Obey the Law^47^ includes six items on a 4-point Likert scale (1 = ‘strongly disagree’; 4 = ‘strongly agree’, α = 0.64). The POOL was calculated as a mean score of all items (M = 2.78, SD = 0.61), with a higher score indicating a higher perceived obligation to obey the law. Cronbach’s alpha was 0.671 in the current study.

### Procedure

Participants were offered course credits to take part in an online study. Wenjuanxin was used as the data collection platform, providing functions equivalent to Amazon Mechanical Turk. The participants were asked to answer the AIS, HEXACO, RC, and POOL questionnaires and then reported demographic information (gender and age). Data were excluded from the analysis if the participants failed to choose the correct answer of the “filter” items (e.g. “Please choose #1 on this question”). A total of 397 questionnaires were collected and 370 were valid (rejection rate = 6.80%).

### Data analysis

SPSS and Amos 26.0 were used for data analysis. Pearson correlation analysis and structural equation model were conducted to test the hypotheses.

## Results

### Results of the correlation analysis

Pearson correlation was used to examine the correlations among the variables. Regarding the effects of demographic variables on academic dishonesty, age was positively correlated with academic dishonesty (r = 0.208, *p* < 0.01). As age increased, people were more likely to conduct various academic misconducts.

Regarding the effects of personality traits on academic dishonesty, as expected and in line with a prior study^9^, we found strong negative correlations between personality and academic dishonesty on all six dimensions. Specifically, academic dishonesty was negatively predicted by honesty-humility (r = − 0.362, *p* < 0.01), emotion stability (r = − 0.119, *p* < 0.05), agreeableness (r = − 0.246, *p* < 0.01), conscientiousness (t = − 0.231, *p* < 0.01), openness to experience (r = − 0.190, *p* < 0.01), and extraversion (r = − 0.185, *p* < 0.01).

Finally, regarding the effect of attitudes towards the rule on academic dishonesty, rule conditionality was positively correlated with academic dishonesty (r = 0.231, *p* < 0.01). It showed that academic misconduct was more acceptable as students scored higher on rule conditionality, consistent with the hypothesis. Contrary to the hypothesis, however, perceived obligation to obey the law/rule was not correlated with academic dishonesty (r = − 0.009, p = 0.864). In addition, POOL was also not associated with five of six personality dimensions nor rule conditionality (r_HH_ = − 0.001, *p* = 0.984; r_EM_ = 0.043, *p* = 0.413; r_EX_ = 0.009, *p* = 0.861; r_AG_ = 0.012, *p* = 0.816; r_CO_ = 0.007, *p* = 0.897; r_RC_ = − 0.016, *p* = 0.761) (see Table 1).

**Table 1:** Descriptive Statistics and Correlations among Variables.

	Mean	SD	1	2	3	4	5	6	7	8	9	10	11	12		13
Age (1)	19.770	3.633	1.000	− 0.183**	− 0.104**	− 0.108**	− 0.174**	.014	− 0.045	− 0.082	0.106*	0.214**	0.194**		0.181**	0.208**
HH (2)	3.231	0.608		1.000	− 0.091	0.146**	0.337**	0.264**	0.095	− 0.172**	− 0.001	− 0.352**	− 0.353**		− 0.314**	− 0.362**
EM (3)	3.328	540			1.000	− 0.090	− 0.069	0.010	0.067	0.097	0.043	− 0.135**	− 0.098		− 0.083	− 0.119*
EX (4)	3.179	0.608				1.000	0.352**	0.263**	0.306**	− 0.035	.009	− 0.173**	− 0.175**		− 0.197**	− 0.185**
AG (5)	3.310	0.525					1.000	0.279**	0.266**	− 0.097	.012	− 0.225**	− 0.241**		− 0.246**	− 0.246**
CO (6)	3.232	0.499						1.000	.268**	− 0.025	.007	− 0.206**	− 0.205**		− 0.250**	− 0.231**
OP (7)	3.293	0.575							1.000	− 0.014	− 0.134**	− 0.166**	− 0.193**		− 0.195**	− 0.190**
RC (8)	3.495	1.082								1.000	− 0.016	0.214**	0.222**		0.239**	0.231**
POOL (9)	2.782	0.615									1.000	.003	− 0.010		− 0.040	− 0.009
Cheating (10)	2.050	1.283										1.000	0.884**		0.841**	0.974**
RM (11)	2.126	1.285											1.000		0.866**	0.951**
Plagiarism (12)	2.225	1.337													1.000	.920**
AD (13)	2.106	1.229														1.000

*AD* Academic dishonesty, *RM* Research misconduct, *RC* Rule conditionality, *POOL* Perceived obligation to obey the law, *HH* Honesty-humility, *EM* Emotion stability, *EX* Extraversion, *AG* Agreeableness, *CO* Conscientiousness, *OP* Openness to experience.

*p < 0.05 (2 tailed), **p < 0.01 (2 tailed).

### Results of the structural equation modeling

**Figure 1:**
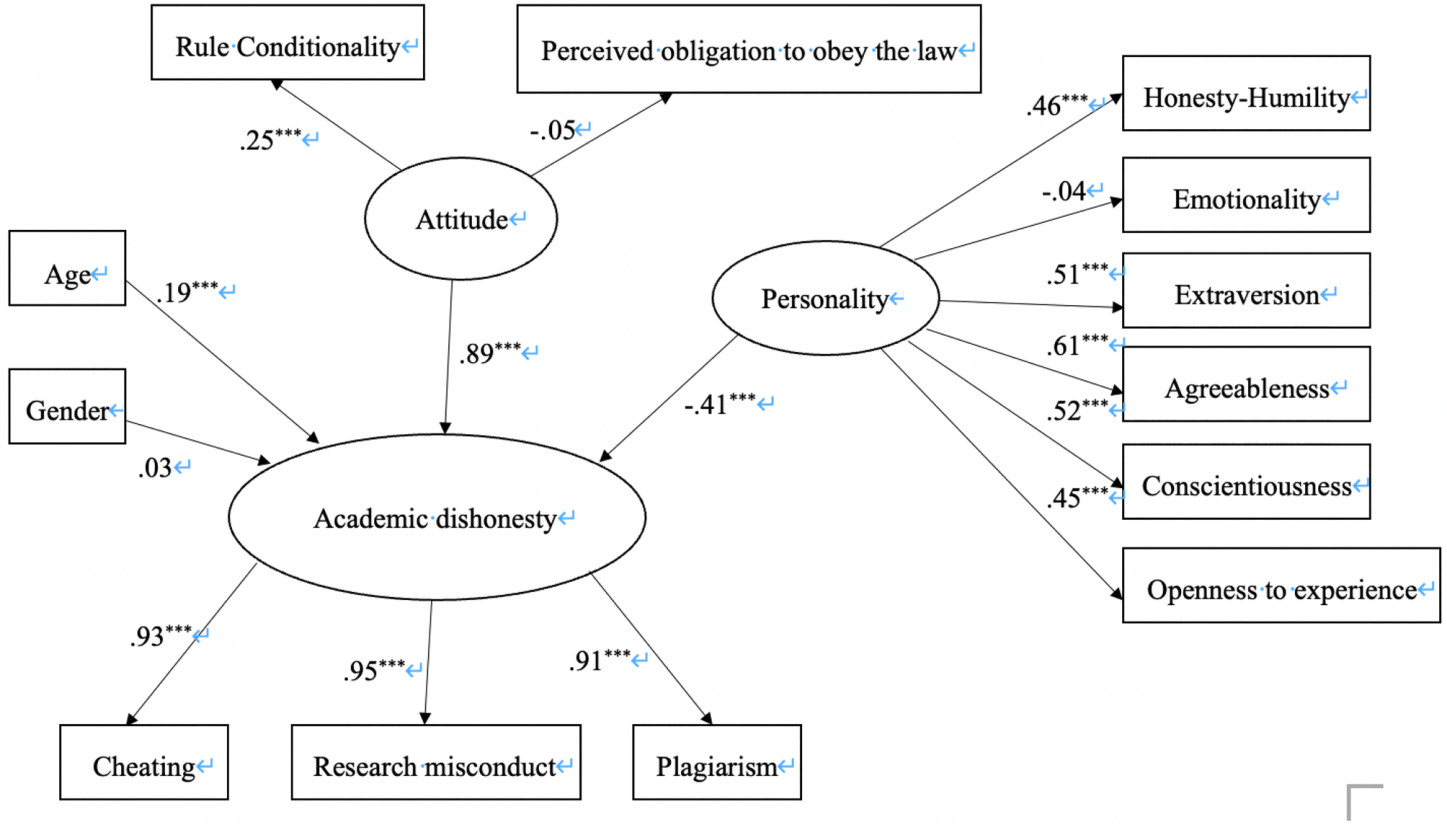
Structural model for determinants of academic dishonesty.

The structural equation models linking the demographic variables (age and gender), the personality variables (HEXACO), and the attitude variables (RC and POOL) were tested. The findings were presented in Fig. 1. The model was examined for the goodness of fit using indices including Chi-square, comparative fit index, and root mean square error of approximation. The results indicated an overall good model fix (χ2 = 180.714, χ2 /df = 2.82, *p* < 0.001; CFI = 0.922; RMSEA = 0.070)^52^.

In general, students’ tendency to engage in academic dishonesty was accounted for by the demographic variables, the personality variables, and the attitude variables. Specifically, consisting to the results of correlation analysis, age but not gender positively predicted academic dishonesty. The HEXACO model negatively predicted academic dishonesty, with five out of the six dimensions being significant except for the emotionality dimension. It indicated that people who scored higher on honesty-humility, agreeableness, conscientiousness, openness to experience, and extraversion were less likely to engage in academic misconduct. In addition, rule conditionality positively predicted academic dishonesty, indicating that individuals who believed in the conditionality of rules were more likely to misbehave at school. These results partially aligned with the hypotheses.

## Discussion

The purpose of this study is to explore the influence of individual factors (personality and people’s general attitudes toward the rules) on academic dishonesty. A survey among university students was conducted, and the results provided evidence that both personality traits and people’s rule orientation had significant effects on their various academic misconducts. The HEXACO model is a relatively recent personality model to expand and replace the Big Five personality model, and its cross-cultural applicability has been verified in many research settings^41^. The current study applied the HEXACO model in predicting academic dishonesty for the first time, and our findings justified its application.

Attitudes towards the rule are manifested in two aspects. Rule conditionality assessed whether individuals would violate relevant regulations and commit deviant behaviors under certain circumstances. Perception of the obligation to obey the law and rules (POOL) assessed individuals’ sense of duty to avoid behaviors that violate regulations, such as academic misconduct. Results of the current study demonstrated that rule conditionality positively predicted academic misconduct; that is, individuals who believed that rules are conditional and could be broken under certain conditions are more likely to engage in various academic transgressions. POOL, however, was not found to be related to academic dishonesty. Previous results showed that Chinese students scored lower on the POOL than American students^53^. POOL is likely an inadequate measure of Chinese students’ sense of obligation and duty to obey the laws and rules. Future research could use a different sample to test the predicting effect of POOL on academic dishonesty, and should also consider revising and refining the POOL measure for cultural research.

Regarding the predictive effect of demographic variables on academic misconduct, only age was found to have a significant positive correlation with academic misconduct. Gender had no effect which is consistent with the previous literature^9,17^.

## Conclusion and implication for future research

The current study examined the HEXACO model and peoples’ attitudes toward the rule to better understand the academic misconduct behaviors among university students. Our findings have important implications for researchers and institutional educators. We demonstrated that in addition to the tractional big five personality factors, honesty-humility is a unique contributor to decreasing academic dishonesty. Recent studies have suggested focusing on integrity as the broadest defense against dishonesty in all spheres of academia^54^. Our research findings encourage university educators and institutional policymakers to pay much attention to this dispositional protector.

Our study also linked law-abiding attitudes with academic behaviors. Legal laws and academic rules share features in regulating people’s behaviors by imposing sanctions. They are quite different from social norms, which is a rather indirect way of behavior regulation. The current study confirmed that rule conditionality plays an important role in predicting academic misconduct of university students. Thus law-abiding education and behavior modification interventions might also be effective in preventing academic dishonesty.

Our results have contributed to the academic dishonesty field, but it is not free from limitations. First, it was a correlational study, meaning it is only possible to speak about relationships but not causal links. Experiments are necessary for future research. In addition, we did not control the participant’s prior academic performance. A previous study showed that students with lower than average performance tend to cheat^55^. Future research should control and study the links in a longitudinal data set. Finally, although the current research only focused on the impact of individual factors on academic misconduct, contextual factors could be considered using multi-level analysis.

## Data Availability

The datasets generated and analyzed during the current study are not publicly available due the present study is a part of a bigger study that has not been completed yet, but is available from the corresponding author on reasonable request.
